# Supplementary material for: MetaRibo-Seq measures translation in microbiomes
Source: Nat Commun. 2020 Jun 29;11:3268. doi: 10.1038/s41467-020-17081-z (PMC7324362; doi:10.1038/s41467-020-17081-z)
Supplement: Supplementary file 10 — Supplementary Data 7 [file 41467_2020_17081_MOESM10_ESM.zip › File2/Confidence_High_Taxonomy/324036_out.krona.html]

Javascript must be enabled to view this page.

members
magnitude
magnitudeUnassigned
count
unassigned
taxon
rank

324036\_out

3

superkingdom
2
3

203691
1
phylum

class
203692
1

136
1
order

family
1
137

157
1
genus

species

SRS018826\_contig\_number\_1272
1
58231

phylum
32066
1

class
1
203490

203491
1
order

family
1
1129771

genus
32067
1

species

SRS097905\_contig\_number\_9014
1
157687

phylum
1
976

1
200643
class

order
171549
1

1
171552
family

genus
838
1

1262925
1

SRS049959\_contig\_number\_35190
species
